# Renal and Hematological Effects of CLCF-1, a B-Cell-Stimulating Cytokine of the IL-6 Family

**DOI:** 10.1155/2015/714964

**Published:** 2015-06-04

**Authors:** Virginia J. Savin, Mukut Sharma, Jianping Zhou, David Gennochi, Timothy Fields, Ram Sharma, Ellen T. McCarthy, Tarak Srivastava, Jos Domen, Aurélie Tormo, Jean-François Gauchat

**Affiliations:** ^1^Renal Research Laboratory, Research and Development, MBRF and Kansas City VA Medical Center, Kansas City, MO 64128, USA; ^2^Kidney Institute, University of Kansas Medical Center, Kansas City, KS 66160, USA; ^3^Section of Nephrology, Children's Mercy Hospital and University of Missouri at Kansas City, Kansas City, MO 64108, USA; ^4^Section of Cardiac Surgery, Children's Mercy Hospital and University of Missouri at Kansas City, Kansas City, MO 64108, USA; ^5^Department of Pharmacology, University of Montreal, Montreal, QC, Canada H3T 1J4

## Abstract

CLCF-1 is a cytokine known for B-cell stimulation and for neurotrophic properties. We have identified CLCF-1 as a potential injurious factor in the human renal disease focal segmental glomerulosclerosis (FSGS). We investigated its effects on renal cells and renal function in *in vitro* and *in vivo* studies. Methods include measurement of the effect of CLCF-1 on phosphorylation of target molecules of the JAK/STAT pathway, on cytoskeleton and cell morphology in cultured podocytes, on albumin permeability of isolated rat glomeruli, and on tissue phosphorylation and urine albumin after acute or chronic CLCF-1 injection. In addition, cell sorting was performed to determine the presence of cells expressing CLCF-1 in spleen and bone marrow of normal mice and the effect of CLCF-1 infusion on splenic B-cell populations. CLCF-1 increased phosphorylation of STAT3 in multiple cell types, activated podocytes leading to formation of lamellipodia and decrease in basal stress fibers, increased glomerular albumin permeability, and increased STAT3 phosphorylation of peripheral blood cells and renal cortex. CLCF-1 increased urine albumin/creatinine ratio in mice and increased B-cell expression of IgG in mouse spleen. We conclude that CLCF-1 has potentially important systemic effects, alters podocyte function, and may contribute to renal dysfunction and albuminuria.

## 1. Introduction

CLCF-1 was originally described in 1999 by subtractive hybridization using a cDNA library constructed from activated Jurkat lymphoma cells [1, 2]. It was found to have neurotrophic activity and was termed neurotropin-1/B-cell-stimulating factor-3 (NNT-1/BSF-3) [2]. It is expressed in lymph nodes and spleen, bone marrow, peripheral blood lymphocytes, ovary, placenta, kidney, pituitary, fetal liver, and other tissues [3]. It can be actively secreted from cells with heteromeric partners including cytokine receptor-like factor-1 (CRLF-1) and soluble ciliary neurotrophic factor receptor *α* (sCNTFR*α*) [4, 5]. CLCF-1 is important in neural differentiation and survival and may serve as a ligand for CNTFR*α* in supporting neural growth [6]. Its partner CRLF-1 may play a role in response to injury [7]. There have been no reports that implicate either of CLCF-1 or CRLF-1 in initiating injury or causing disease.

We have been studying human focal segmental glomerulosclerosis (FSGS) for more than 20 years [8–15]. FSGS describes a histopathological lesion characterized by loss of podocyte foot process and segmental glomerular scarring. Clinical manifestations of FSGS include both steroid-sensitive and steroid resistant nephrotic syndrome. Many patients progress to renal failure. Genetic FSGS involves mutations in proteins expressed by podocytes. The slit diaphragms are highly specialized intercellular junctions between podocytes that provide the final barrier to protein filtration [16–21]. In the majority of patients with FSGS, no genetic abnormalities have been identified. After renal transplantation, FSGS recurs in 30 to 50% of patients [11, 21–23]. We and others have shown that plasma or serum of such patients impairs glomerular barrier function and affects the morphology of cultured immortalized podocytes and have employed* in vitro* assays to direct efforts to identify molecules that may lead to FSGS and its posttransplant recurrence [8, 24–26]. We have used affinity chromatography and mass spectrometry to identify CLCF-1 as a potential plasma permeability factor in FSGS [15].

The role of CLCF-1 and related cytokines in control of the function of mature cells has not been studied exhaustively. The series of studies described here document the presence of cells that express CLCF-1 in mouse bone marrow as well as the effect of CLCF-1 on differentiation of B cells recovered from the spleen after CLCF-1 infusion and on relevant signal pathways in circulating blood cells, renal cortex, glomeruli, and tubules, and on cultured podocytes. Studies of the glomerular barrier* in vitro* and of albuminuria in mice confirm the relevance of these effects to renal function. The results are consistent with our postulate that CLCF-1 may contribute to human renal disease, specifically FSGS in patients with recurrence after renal transplant.

## 2. Methods and Materials

### 2.1. Reagents and Solutions

Recombinant human CLCF-1 (rhCLCF-1) and monoclonal anti-CLCF-1 antibody were obtained from R&D Systems, Minneapolis, MN. Buffers and media were prepared using chemicals obtained from Sigma-Aldrich (St. Louis, MO). These reagents were stored following the vendors' guidelines. Working solutions were prepared in medium containing 5% BSA. The JAK2 inhibitor BMS-911543 was obtained from ChemieTek, Indianapolis, IN. Stock solutions were prepared and stored following instructions of suppliers/manufacturers.

### 2.2. Animals

Studies were carried out using protocols approved by the Institutional Animal Care and Use Committee (IACUC), Safety Subcommittee, and the R&D Committee at the Medical College of Wisconsin or the VA Medical Center, Kansas City, MO. All animals were maintained at AAALAC-approved facilities at 68–78°F ambient temperature and 30–70% humidity under 12/12-hour light and dark cycles with unrestricted access to food and water.

#### 2.2.1. Mice for Studies of JAK and STAT Phosphorylation and Albuminuria

The 1–12-week-old male C57B6 mice 1 (Charles River Laboratories, Indianapolis, IN) were used to study effects of intraperitoneal (IP) injection of CLCF-1, 1–10 *µ*g/kg/day for up to 3 days, or infusion of CLCF-1 by osmotic minipump at a dose of 10 *µ*g/kg/day for up to 28 days. Blood samples were drawn by venipuncture at intervals after injection and kidneys were obtained at sacrifice under anesthesia. Urine was collected by spontaneous voiding.

#### 2.2.2. Mouse Bone Marrow and Spleen Cells Analyzed for CLCF-1 Expression

Cells were obtained from bone marrow and spleen of FVB mice (Charles River Laboratories) after euthanasia under anesthesia. Cells were stained with rabbit anti-CLCF-1 polyclonal serum (Santa Cruz Biotechnology NNT-1/BSF-3 (FL-225)) which was raised against a human CLCF-1 peptide and cross-reacts with mouse and rat protein. Normal rabbit IgG (catalog #: sc2027) was used as an isotype control. Detection was performed with goat-anti-rabbit-FITC (Pharmingen 554020). Fixation and permeabilization solution from eBioscience was used as recommended. The data were analyzed using Becton-Dickenson FACSCalibur and FlowJo single cell analysis software.

#### 2.2.3. Rats for Isolation of Glomeruli for Permeability Studies

Adult male Sprague-Dawley rats (7-8 weeks old, 200–250 g body weight) were obtained from Harlan, Madison, WI. Glomeruli were isolated from renal cortex of kidneys removed immediately after euthanasia under anesthesia. Details of glomerular isolation are described below.

### 2.3. Isolation of Mouse Bone Marrow or Spleen Cells for Expression of CLCF-1 or IgG1

#### 2.3.1. Expression of CLCF-1 by Mouse BM Cells

Cells were retrieved from bone marrow of normal mice using gradient density centrifugation. They were stained with antibodies for CD3, CD45R, TER119, Gr-1, and CD11b. They were analyzed using 4-color FACSCalibur (BD Bioscience) flow cytometer.

#### 2.3.2. Expression of IgG in Splenocytes after Infusion of CLCF-1

Mouse spleen mononuclear cells were isolated by Ficoll-Histopaque gradient density centrifugation and incubated with Fc Block (BD Biosciences) in PBS 0.5% BSA. Cells were washed and stained with a combination of APC-labeled rat anti-CD19 mAb and Alexa 488-labelled rat anti-IgG1 (both from BD Bioscience) for 30 min on ice. Fluorescence was analyzed using a FACSCalibur (BD Biosciences) flow cytometer [27]. Results were expressed as percentage of B cells expressing IgG1 (CD19+ IgG1 double positive cells) in whole spleen.

### 2.4. Human Peripheral Blood Samples

#### 2.4.1. Human Subjects

The Institutional Review Board of the University of Kansas Medical Center and of Medical College of Wisconsin or NIDDK, NIH (Kopp), approved all studies of human specimens obtained during the tenure of the authors at the respective institutions. Specimens collected specifically for this study were obtained after written informed consent from the donors. The diagnosis of FSGS was confirmed by renal biopsy in the native kidney. Recurrent FSGS after renal transplantation was defined by nephrotic range proteinuria in the early posttransplant period and, in most cases, by biopsy of the allograft showing podocyte foot process effacement.

#### 2.4.2. Human Serum and Plasma Specimens for Measurement of CLCF-1

Serum or plasma was obtained from samples of peripheral blood obtained from normal volunteers and FSGS patients with proteinuria recurrence following kidney transplant. Plasma concentrations of CLC-1 were determined using immunocapillary electrophoresis. This technique is linear across concentrations from 200 to 10 pg/mL and permits measurement of concentrations as low as 10 pg/mL.

### 2.5. Glomerular Albumin Permeability (*P*_alb_) Assay Using Glomeruli Isolated from Normal Rats

Glomeruli from Sprague-Dawley rats were used to study changes in glomerular filtration barrier characteristics using an* in vitro* assay established in our laboratory [28]. Briefly, rat glomeruli were isolated and suspended in a physiological solution (pH 7.4) containing bovine serum albumin (BSA) 5 gm/dL (isolation/incubation buffer). Isolated glomeruli were treated with control or test agents for 15 minutes at 37°C. A video-image was recorded and medium was changed to 1% BSA while additional images were recorded. The change of medium produced an oncotic gradient across the glomerular capillary wall and caused fluid influx into the capillaries and an increase in glomerular volume. Glomerular volume was estimated from the geometric mean of 4 glomerular diameters measured at 45° angles. The change in volume (Δ*V*) of each glomerulus in response to the oncotic gradient was calculated as Δ*V* = (*V*_final_ − *V*_initial_)/*V*_initial_ × 100%. The increase in glomerular volume (Δ*V*) was used to calculate convectional albumin permeability (*P*_alb_) which describes the movement of albumin consequent to water flow. In normal glomeruli, *P*_alb_ is zero and the ratio of Δ*V* of control and experimental glomeruli are equal. *P*_alb_ increases with loss of the permeability barrier and the ratio of Δ*V* of experimental glomeruli to Δ*V* of experimental glomeruli falls proportionately. In each experiment, 5 glomeruli from each experimental condition for each rat were measured and the average was used in further analyses. Experiments were repeated in triplicate.

### 2.6. Western Blotting and Determination of STAT3 Phosphorylation

Tissue was homogenized in lysis buffer containing Sigma Fast Protease Inhibitor (S8820, 119K8203 Sigma-Aldrich, St. Louis, MO) and phosphatase inhibitors (P5726 and P0044, Sigma-Aldrich) using a sonicator and the lysate was centrifuged at 12,000 g for 5 minutes. Total protein was determined using a kit based on Lowry's assay (Bio-Rad, Hercules, CA). The supernatant was frozen at −70°C. Total protein was electrophoresed by SDS-PAGE using TGX gels (Bio-Rad) followed by electrotransfer to PVDF membrane and detection using specific primary antibodies. Rabbit anti-pSTAT3 (Tyr705 D3A7, Cell Signaling catalog # 9131, 1 : 1000 dilution) was used in 5% BSA TBST. Mouse anti-*β*-actin (Sigma catalog # A5441, 1 : 10000) was used in 5% dry milk in TBST. HRP-conjugated secondary antibodies for pSTAT3 (Tyr 705) and *β*-actin were goat anti-rabbit HRP conjugate (Bio-Rad, catalog # 1705046, 1 : 10,000 dilution) and goat anti-mouse HRP conjugate (Bio-Rad, catalog # 170-5047, 1 : 10,000), respectively. ECL Prime Western Blotting Detection reagent (GE Health Sciences, Piscataway, NJ) was used for chemiluminescence reaction and images were obtained using Kodak Gel Logic 2200 imaging system (Carestream Health, New Haven, CT). Image intensity data were normalized by loading control *β*-actin. Normalized intensity ratios were used to prepare bar graphs shown. The effect of CLCF-1 on STAT3 (Tyr 705) phosphorylation was measured. Normalized intensity ratios were used to prepare bar graphs shown.

### 2.7. Cell Culture and Confocal Microscopy

Immortalized murine podocytes [29] were grown on collagen coated glass coverslips at 33°C to subconfluence and then transferred to 37°C to permit differentiation. They were treated with CLCF-1 or other reagents, fixed in 4% paraformaldehyde in phosphate buffered saline (PBS) for 15 minutes at room temperature, and then washed with PBS. After fixation, cell membranes were permeabilized with 0.1% Triton-X100 in PBS (10 minutes) and 0.05% Tween-20 (10–15 minutes). The actin cytoskeleton was stained using Alexa Fluor 568 Phalloidin, Invitrogen, A12380. Nuclei were stained with Hoechst 33342. Cover slips were mounted in 5% *n*-propyl gallate in buffered glycerol (glycerol : PBS, 9 : 1). Cells were viewed using a Leica DMI 4000 B confocal microscope. *Z*-sections 0.25 *µ*m in thickness were obtained for analysis. Images were taken using a 40x objective lens and a 561 nm laser. Laser intensity, gain settings, scaling, individual section depth, and pinhole settings were kept constant through the entire experiment so that intensities can be compared directly. Images were analyzed using Image J software. Parameters measured included cell perimeter and area, total intensity of actin, and presence and density of peripheral actin arcs as well as proportion of the cell periphery that was made up of lamellipodia. The number, thickness, and parallel configuration of basal actin fibers were assessed semiquantitatively.

### 2.8. Renal Histology

Renal cortex was examined by light microscopy after infusion of CLCF-1 for 28 days. Tissue was fixed in formalin, embedded in paraffin, and stained with Jones stain and counterstained with H and E. Additional sections were stained for pSTAT Tyr705 using a phospho-Tyr705-specific Stat3 antibody from Cell Signaling (# 9145).

### 2.9. Statistical Analyses

Values of studies with 2 groups were compared using Student's *t*-test. In studies in which more than 2 groups were compared, ANOVA was used. *P* < 0.05 was accepted as significant.

## 3. Results

### 3.1. Demonstration of CLCF-1 Producing Cells in Mouse Bone Marrow by Flow Cytometric Analysis

**Figure 1:**
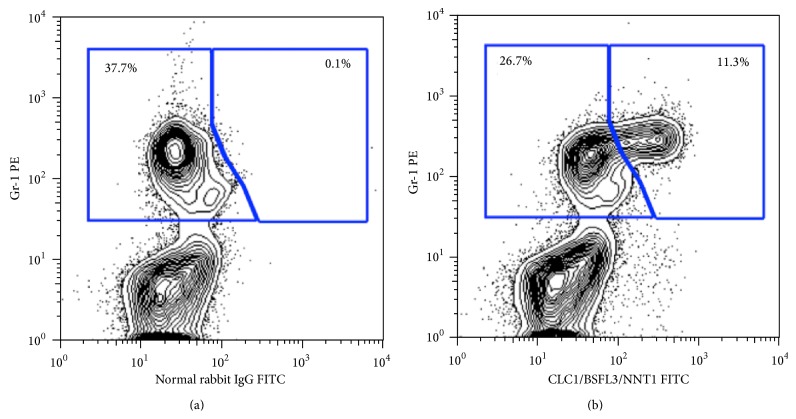
(a) Expression of CLCF-1 in bone marrow of FVB mouse. Cells were isolated from bone marrow (BM) and subjected to flow cytometry. Approximately 11% of BM cells stained with anti-CLCF-1. These cells were also positive for the myeloid differentiation antigen GR-1 but did not express markers for B or T lymphocytes (see text). A representative experiment is shown.

Studies were performed to demonstrate potential hematopoietic sources of CLCF-1 in the mouse using intracellular staining and flow cytometry. Ly-6G, formerly known as the myeloid differentiation antigen Gr-1, is a GPI-anchored protein. In the bone marrow, the level of antigen expression is directly correlated with granulocyte differentiation and maturation. It is also transiently expressed on monocytes in the bone marrow. In the periphery, Ly-6G is expressed predominantly on neutrophils. A set of representative plots are shown to compare the normal rabbit IgG and immune serum. A subset of hematopoietic cells showed specific staining with the anti-CLCF-1 polyclonal serum. These cells were negative for CD3, CD45R, and TER119 (not shown) but positive for Gr-1 (shown) and CD11b (not shown). Approximately 10% of bone marrow cells and 1-2% of splenocytes stained with anti-CLCF-1. The CLCF-1 expressing cells appear to be myeloid cells, most likely neutrophils, and do not include T or B lymphocytes or erythroid cells. See Figure 1(a). LPS or ConA stimulation of splenocytes did not result in increased numbers of CLCF-1 positive cells (data not shown). These findings suggest that hematopoietic cells producing CLCF-1 are present in the mouse in the basal state and that they are not increased by immune stimulation.

### 3.2. Effect of Injection of CLCF-1 on B-Cell Expansion

**Figure 2:**
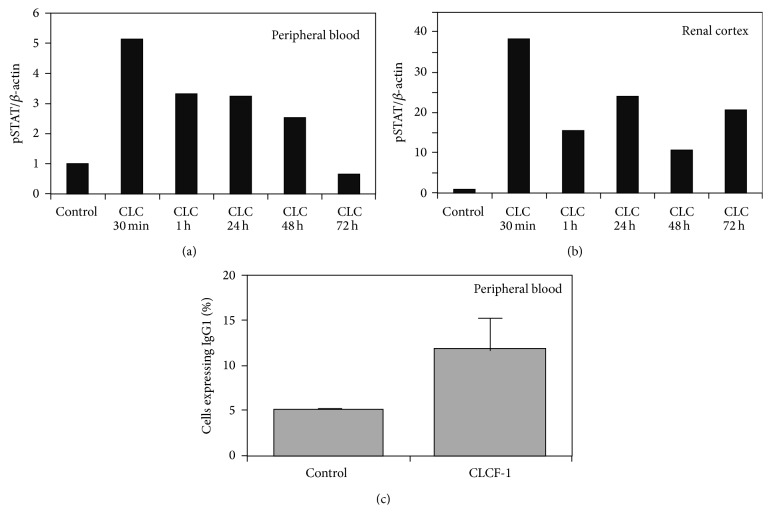
Total pSTAT3 in (a) peripheral blood cells and (b) renal cortex was determined by Western blot analysis of lysates as described in Methods and Materials. Values are expressed as the ratio of pSTAT to *β*-actin. Results of a single experiment are shown. Note that the maximum increase in pSTAT3 is nearly 8-fold greater in kidney cortex compared to blood cells. (c) Mouse spleen mononuclear cells were isolated and analyzed as described in Methods and Materials. Results are expressed as percentage of B cells expressing IgG1 (CD19+/IgG1+). Mean of 3 determinations in each of the 2 mice was used to calculate values, expressed as mean ± standard deviation. CD19+/IgG1+ cells were increased after infusion of CLCF-1. ^*∗*^ *P* < 0.01.

The percentage of splenic B cells that expressed IgG was increased after CLCF-1 infusion by minipump for 28 days. The result of duplicate studies is shown in Figure 2(a). No data for earlier time points are available. This finding is expected from prior understanding of CLCF-1 as a B-cell stimulating cytokine.

### 3.3. Phosphorylation of Peripheral Blood Cells and Renal Cortex of Mice after Acute Injection or Chronic Injection of CLCF-1

Acute intraperitoneal injection of CLCF-1 led to phosphorylation of STAT3 in peripheral blood cells. pSTAT was present within 15 minutes of injection and peaked within 1 hour. Increased pSTAT3 persisted for 72 hours after a single injection but was decreasing toward baseline at the end of this period, Figure 2(b). pSTAT3 was also increased in renal cortex at the same time intervals after an injection of CLCF-1. See Figure 2(c). Findings confirm that CLCF-1 affects cells outside its traditionally recognized targets of immune cells. Of note, the signaling effect of CLCF-1 is greater in magnitude and persists longer in renal cortex than it does in peripheral blood cells.

### 3.4. Effects of CLCF-1 on Glomerular *P*_alb_ and Dependence on JAK2 Phosphorylation

**Figure 3:**
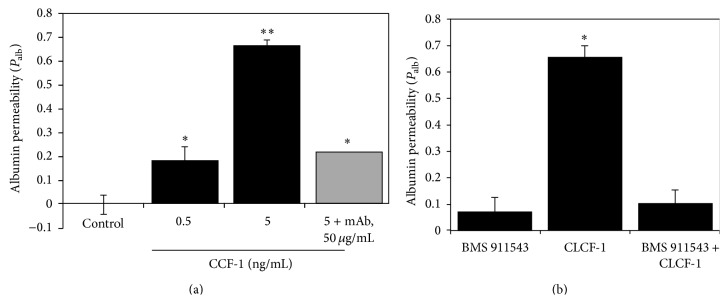
(a) Isolated rat glomeruli were incubated with the indicated concentrations of CLCF-1 for 15 minutes at 37°C. Anti-CLCF-1 antibody abrogated this effect with maximum effect at antibody concentration of 50 *µ*g/mL. *N* = 10 at each concentration. Values are mean ± SEM. ^*∗*^ *P* < 0.05, ^*∗∗*^ *P* < 0.001 versus control. (b) JAK inhibition of CLC effect on *P*_alb_. Isolated rat glomeruli were incubated with 10 ng/mL CLCF-1 for 10 minutes at 37°C either with or without pretreatment by 5 nM JAK2 inhibitor BMS911543 for 15 min at 37°C. Additional glomeruli were treated with BMS911543 alone. CLCF-1 increased *P*_alb_ was blocked by pretreatment with BMS911543. ^*∗*^ *P* < 0.001 versus BMS911543 alone or CLCF-1 after BMS911543.

Recombinant human CLCF-1 (rhCLCF-1, CLCF-1) at subnanomolar concentrations of 0.05–100 ng/mL increased glomerular *P*_alb_ in a dose-dependent manner. A significant increase in *P*_alb_ was evident at CLCF-1 concentrations as low as 0.05 ng/mL. A maximal increase was observed at 5 ng/mL (*P* < 0.001). Responses to concentrations of 0.5 and 5 ng/mL are shown in Figure 3(a). Plasma from patients with recurrent FSGS typically increases *P*_alb_ to between 0.7 and 0.8 [8, 10]. Anti-CLCF-1 monoclonal antibody blocked the increase in *P*_alb_ caused by CLCF-1 (Figure 3(a)). For the studies shown, *P*_alb_ was measured after glomerular incubation with CLCF-1 (5 ng/mL) or with CLCF-1 and anti-CLCF-1 antibody (50 mg/mL) for 15 min. Anti-CLCF-1 mAb also markedly diminished the *P*_alb_ response to plasma of patients with recurrent FSGS to as little as 5% of uninhibited values (data not shown). Inhibition of *P*_alb_ activity of CLCF-1 by mAb was specific as evidenced by the fact that neither preimmune rabbit IgG (control) nor antibodies to TNF, TGF, or IL-6 protected glomeruli from the effects of CLCF-1 (data not shown). These observations document the capacity of CLCF-1 to impair the glomerular barrier to albumin.

To test the hypothesis that the effect of CLCF-1 depends on JAK2 activation we incubated glomeruli with the JAK2 inhibitor BMS911543 prior to addition of CLCF-1. This inhibitor is specific for the JAK2 isoform. BMS911543 markedly decreased the effect of CLCF-1 on *P*_alb_; see Figure 3(b). The inhibition of CLCF-1 effect on *P*_alb_ is consistent with the hypothesis that JAK2 phosphorylation is a necessary initial step in control of glomerular permeability.

### 3.5. Effects of CLCF-1 on Actin Cytoskeleton of Cultured Podocytes

**Figure 4:**
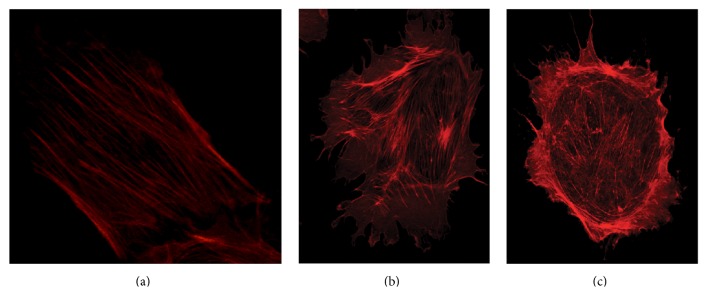
Immortalized podocytes were incubated with either (a) vehicle (b, c) 10 ng/mL CLCF-1 for (b) 15 minutes or (c) 30 minutes. Subsequently, cellular actin was stained as described in Methods and Materials and 0.25 *µ*m sections were visualized by confocal microscopy. Note parallel actin bundles in central portion of control cell (a), compared to the decrease in intensity of central actin bundles and loss of parallel pattern in the cells treated with CLCF-1 (c). Lamellipodia are present in nearly the entire circumference after 30 minutes of incubation (c).

Incubation with CLCF-1 for up to 1 hour caused marked changes in the configuration of the actin cytoskeleton of cultured murine podocytes. Changes progressed with duration of incubation and were concentration dependent. Specifically, the intensity and ordered configuration of stress fibers in the central part of the cells diminished. The number and extent of lamellipodia increased as did the extent and intensity of actin arcs associated with lamellipodia. Lamellipodia, measured as percent of the cell circumference occupied, increased from 21 ± 7% to 82 ± 7% after 1 hour of incubation. Cell area and total actin intensity did not change. These changes are consistent with activation of the cell toward a more motile phenotype that may be more vulnerable to detachment under mechanical or metabolic stress. Representative cell images of control podocyte and of cells after incubation for 15 and 30 minutes are shown in Figures 4(a), 4(b), and 4(c).

### 3.6. Effects of Infusion of CLCF-1 on Phosphorylation of Cells of Mouse Renal Cortex and on Mouse Albuminuria

**Figure 5:**
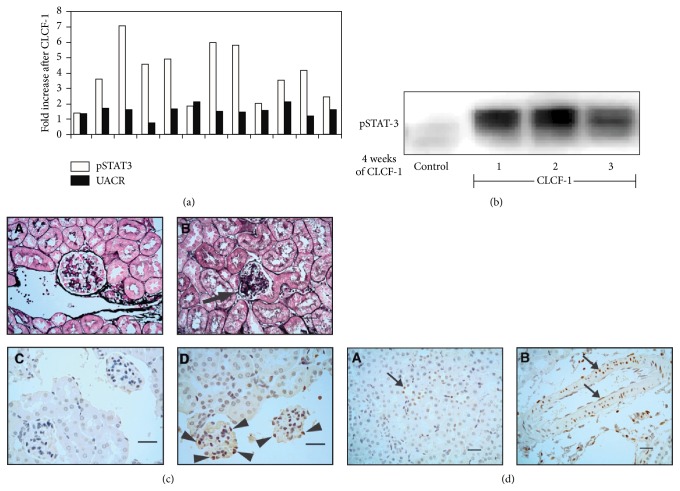
(a–d) Effect of acute injection or 28-day infusion of CLCF-1 on urinary albumin creatinine ratio (UACR) and renal histology and pSTAT3 expression in mice. (a) Renal cortical pSTAT3 and urine albumin creatinine (UACR) ratio 24 hours after a single injection of CLCF-1. Results for one experiment with 12 individual mice are shown as ratio of postinjection values to control values for pSTAT3 and postinjection to preinjection values for individual mice for UACR. pSTAT3 increased to 3.0 ± 1.8 (95% confidence interval 2.8 to 5.1) and UACR increased by 1.62 ± 0.37-fold versus preinjection level (95% confidence interval 1.38 to 1.85). Values are mean ± SD, *N* = 12. (b) CLCF-1 infusion increases pSTAT3 expression in renal cortex. After 4 weeks of infusion of CLCF-1 or vehicle (control) by minipump, mouse renal cortex was harvested and lysates were examined for pSTAT3 expression by Western blot. Results are shown for cortex of 3 individual mice that received CLCF-1 and one control mouse. (c) Histology of mouse renal cortex after 4 weeks of CLCF-1 or vehicle (control) infusion. Kidneys were harvested from treated and control mice, and histologic sections were prepared and stained as described in Methods and Materials. (A) Control mouse (Jones silver stain). Glomeruli showed normal cellularity and mesangial matrix without evidence of sclerosis (>150 glomeruli counted). Scale bar = 25 *µ*m. (B) CLCF-1 infusion (28 days; Jones silver stain). Renal cortex after 28 days of CLCF-1 infusion. Rare glomeruli (2 in >150 glomeruli counted) showed segmental sclerosis, with collapse of capillaries and segmental increase in matrix (arrow). Scale bar = 25 *µ*m. (C) Anti-pSTAT3 staining of renal cortex from control treated mouse. No staining was evident. Scale bar = 25 *µ*m. (D) Anti-pSTAT3 staining of renal cortex from mouse treated for 28 days with infusion of CLCF-1. Occasional glomerular epithelial cells showed pSTAT3 staining (arrowheads). Scale bar = 25 *µ*m. (d) Anti-pSTAT3 staining of renal cortex from mouse treated for 28 days with infusion of CLCF-1. Focal staining of tubules (A), as well as smooth muscle and endothelial cells (B), is indicated by arrows. No staining was observed in sections of control mouse kidneys. Scale bar = 50 *µ*m.

A single injection of CLCF-1 significantly increased both renal cortical pSTAT and urine albumin/creatinine ratio (UACR). Results of a representative experiment are shown in Figure 5(a). In this experiment, both pSTAT3 and UACR increased significantly. Chronic infusion of CLCF-1 for 28 days increased the UACR values from control of 0.20 ± 0.05, *N* = 10, to post-28-day infusion of 0.57 ± 0.46. *N* = 4 (*P* < 0.02).  Figure 5(b) shows results of Western blots for pSTAT3 in control and 3 individual mice after 28 days of SLSF-1 infusion. Immunohistochemistry showed increased pSTAT3 in cells of the glomerulus, renal tubules, and renal arterioles. Segmental lobular solidification with mesangial expansion and obliteration of capillaries was present in rare glomeruli after infusion but not in control kidneys. pSTAT3 was present in nuclei of glomerular cells after treatment with CLCF-1 but not in those treated with vehicle. Results of histology and immunohistochemistry are shown in Figures 5(c) and 5(d). Taken together, these results confirm that CLCF-1 activates pSTAT3 in glomerular and other renal cells and increases albuminuria.

## 4. Discussion

### 4.1. CLCF-1, a Member of the IL-6 Family of Cytokines

CLCF-1 has a predicted molecular weight that is 22 kDa. It shares 19–27% homology with other IL-6 family members. It is predicted to contain *α*-helices and has 1 potential N-linked glycosylation site. Alternative names include cardiotrophin-like cytokine 1 (CLC-1), B-cell stimulatory factor-3 (BSF3), and novel neurotrophin-1 (NNT-1) [30]. CLCF-1 maps to chromosome 11q13.3 [2, 3]. It appears to be secreted efficiently only with a partner such as cytokine receptor-like factor-1 (CRLF-1) or soluble ciliary neurotrophic factor receptor *α* (sCNTFR). It may associate with CRLF-1 in the circulation and many commercially available preparations for recombinant CLCF-1 are supplied as the compound cytokine CLCF-1/CRLF-1. CLCF-1, like other members of the interleukin-6 family, is involved in cell signaling through phosphorylation of glycoprotein 130 (gp130) [5]. It activates a complex receptor consisting of gp130, ciliary neurotrophic factor receptor (CNTFR*α*), and leukemia inhibitory factor receptor (LIFR*α*) [4]. An alternative receptor has been detected on B cells by the use of labeled CLCF-1 but has not been completely characterized [31]. The requirements for interactions between CLCF-1 and related cytokines and specific receptors are under active investigation. Binding partners of these cytokines and interactions with receptor proteins may be cell-type specific. For instance, IL-27-*α* deficient cells can be activated by a heterodimer composed of CRLF-1 and p28 (p28/CRLF-1); this heterodimer activates and induces plasma cell differentiation and IgM, IgG2c, and IgG1 production [27]. Additionally, the compound cytokine CLCF-1/CRLF-1 acts only through the canonical receptor for CLCF-1 (gp130-LIFR*β*-CNTFR*α*), while CNTF can activate cells through an alternate receptor complex gp130- LIFR*β*-IL6R when CNTFR*α* is absent and IL6R is present [32].

CLCF-1 and related molecules are known for their trophic effects on neurons and other cells during development. CLCF-1 is required for motor neuron development and mice lacking it die shortly after birth because they cannot suckle [6, 33]. Additional targets of CLCF-1 action include increased ACTH secretion by corticotroph AtT-20 cells and murine pituitary tissue which is blocked by suppressor of cytokine signaling (SOCS)-3 [34], a significant role in control of branching during fetal lung development [35], and a role in diminishing fibrosis in bleomycin induced lung injury [7].

CRLF-1 has been found in the immature kidney and appears to affect renal development [36]. A case of renal dysplasia associated with cold sweating syndrome and potential deficiency of CLCF-1/CRLF-1 has been reported [37]. Human disease related to inactivation of CLCF-1 leads to autonomic dysfunction in the Crisponi syndrome, also termed cold sweating syndrome [30]. Signaling mediated by CLCF-1 appears to depend primarily on activation of JAK/STAT pathways. Modulation of these pathways includes negative regulation via SOCS [1, 34] as well as activation by specific receptors. Examples of studies of CLCF-1 receptor activation and signaling include phosphorylation of gp130, LIFR-*β*, and STAT3 in human neuroblastoma cells and activation of NF*κ*B and SRE reporter constructs [3]. In addition CLCF-1 may form an active heterodimer with its secretory partner CRLF-1 [5]. This heterodimer activates B cells but, paradoxically, we have found that it blocks the effect of CLCF-1 on glomeruli or podocytes [38]. Overexpression of CLCF-1 in transgenic mice, under control of the apolipoprotein E promoter, led to B-cell hyperplasia with particular expansion of the mature follicular B-cell subset in the spleen and the prominent presence of plasma cells. Mice showed elevated serum levels of IgM, IgE, IgG2b, IgG3, anti-dsDNA Abs, and amyloid A. They produced high amounts of Ag-specific IgM, IgA, and IgE and low amounts of IgG2a and IgG3 [39].

#### 4.1.1. JAK/STAT Pathway and Transcription

CLCF-1 and related cytokines affect cell function through phosphorylation of molecules of the JAK/STAT pathway. We have found that JAK2 and STAT3 are the predominant isoforms in glomeruli and podocytes of mice and rats (unpublished data). STAT3 effects appear to be determined by posttranslational modifications, dimerization, and nuclear translocation. Activation of STAT3 involves phosphorylation at tyrosine 705 (Y705), nuclear translocation, and binding to interferon-*γ* activated sequences for transcription [40–42]. In addition, phosphorylation of serine (S) residues in STATs (S727 in STAT3) may affect STAT translocation in a variety of cell specific ways [43]. STAT3 activation may also occur by serine phosphorylation without tyrosine phosphorylation [40]. Acetylation of STAT3 is responsible for its function as a negative regulator of autophagy [44, 45] and it contributes to oxidative changes in diabetic nephropathy [46]. The range of STAT3 effects remains to be fully defined.

#### 4.1.2. CLCF-1 and the Kidney

CLCF-1 has been suggested as a regulator of kidney development. CRLF-1 is enriched in the ureteric bud and the CLCF-1/CRLF-1 complex caused phosphorylation of STAT3, a reaction typical of mesenchymal-to-epithelial conversion. Incubation of rat metanephric mesenchyme with CLCF-1/CRLF-1 (3 nM) induced structures expressing glomerular and tubular markers [36]. A transgenic mouse with overexpression of CLCF-1 manifested increased B-cell antibody production and increased level of serum amyloid A, as cited above. In addition, these mice developed nonamyloid mesangial deposits that contained IgM, IgG, and C3 and showed a distinctive ultrastructure similar to that of immunotactoid glomerulopathy. No mention of proteinuria is made in the description of this phenotype [39]. We identified activation of STAT3 in cells of glomeruli, renal tubules, and blood vessels after infusion of CLCF-1 as shown above.

### 4.2. CLCF-1 and FSGS

We have identified CLCF-1 in the plasma of patients with FSGS who experience recurrence of proteinuria and renal disease in the allograft after transplantation. Identification was based on the results of LC-MS/MS of plasma after affinity purification using galactose coated Sepharose beads [47]. A single peptide unique to CLCF-1 was identified in the active plasma fraction of two FSGS patients and was not identified in pooled plasma from normal donors. No other cytokine was identified in plasma fractions of patients or normal controls. These findings provided the rationale for testing the activity of CLCF-1 as a regulator of podocyte and glomerular function and as an inducer of proteinuria. The current investigations verify that rCLCF-1 shows activity comparable to that of FSGS plasma in increasing glomerular *P*_alb_ during* in vitro* testing. The permeability activity was blocked by a monoclonal anti-CLCF-1 antibody or by a specific JAK2 inhibitor. These findings are consistent with the interpretation that JAK phosphorylation, such as that caused by interaction of CLCF-1 with its receptor complex, is sufficient to alter glomerular function. In addition, CLCF-1 activated cultured murine podocytes as evidenced by altered cytoskeleton and increased pSTAT3. CLCF-1 also led to an increase in albuminuria after it was injected into mice. These are the first findings that suggest that CLCF-1 regulates mature cells of nonhematological organs and that activation of its receptors may contribute to disease.

### 4.3. Role of CLCF-1 in FSGS and Its Recurrence

The results presented here implicate CLCF-1 as a potential “circulating factor” in recurrent FSGS. CLCF-1 is present in the fraction of plasma of affected FSGS patients that carries permeability activity. It acts promptly to initiate signaling via the JAK/STAT pathway in glomerular podocytes as well as in systemic cells. It alters the morphology of cultured podocytes and the barrier function of glomerular capillaries. Glomerular and podocyte responses are consistent with a direct effect of CLCF-1 to alter function in a manner that leads to proteinuria, the first sign of FSGS. The degree of albuminuria/proteinuria in the mice studied was lower than it is in classical human FSGS. This may be because mice are resistant to developing proteinuria and/or because pathways that are activated in renal disease differ in mice and humans. It is also possible that, since the recombinant reagent used is made in a bacterial system and lacks glycosylation, it may have lower potency than that which is expressed in mammalian cells. Finally, it is possible that additional plasma components are required to cause maximal proteinuria and to result in renal disease that mimics human FSGS. Such plasma components might include antibodies, lipoproteins, or other cytokines or proteins that are also increased by CLCF-1 or by substances generated by the kidney during injury.

As anticipated from the known properties of CLCF-1, the B-cell population producing IgG is expanded after infusion of CLCF-1 into mice. B-cell responses to CLCF-1 may also be important in progression of FSGS to fibrosis and renal failure. A recent report documents elevated concentrations of a number of autoantibodies in the plasma of patients with recurrent FSGS [48]. Targets of these antibodies include proteins expressed by podocytes. It may be that CLCF-1 contributes to B-cell activation that, in turn, leads to generation of these and other antibodies. Rituximab, an anti-B-cell antibody that is useful in autoimmune diseases including membranous nephropathy, has reduced proteinuria in some patients with FSGS including those with recurrence after transplantation. Rituximab has been proposed to act directly to protect podocytes [49] and this effect, as well as decreased antibody synthesis, may be important in FSGS. Thus, CLCF-1 may play a dual role in the pathogenesis of recurrent FSGS, first, by signaling that directly alters podocyte function and, second, by activating B cells and enhancing antibody production.

Unfortunately, currently available assays are not sufficiently sensitive to permit measurement of CLCF-1 in patient samples. Preliminary studies suggest that normal concentrations may be only a few pg/mL and that concentrations in some FSGS patients may be over 100 pg/mL (unpublished data). There is no information regarding potential urinary excretion of CLCF-1 or potential elevation in other disease states. Our findings regarding *P*_alb_ activity suggest kinetics similar to those of immunoglobulins and it is possible that CLCF-1 is bound to plasma proteins or other molecules. *P*_alb_ activity is relatively constant over many months or years and does not vary with filtration rate or degree of proteinuria. If CLCF-1 is indeed directly related to this activity, then it too may remain stable over long periods. We are actively investigating these relationships and the potential clinical utility of CLCF-1 measurements. If an active role for CLCF-1 in glomerular injury is confirmed, therapy to block its effects might induce remission or arrest of progression in FSGS. Therapy might include use of humanized antibodies to CLCF-1 itself, soluble receptors, or cytokine traps to prevent receptor activation. Therapy that blocks the effects of CLCF-1 may provide targeted protection without adverse effects since no essential role of CLCF-1 has been identified after fetal development. Alternatively, inhibition of JAK/STAT activation using currently available small molecules may be protective and have an acceptable side-effect profile.

## 5. Summary and Conclusions

CLCF-1 is expressed in cells of bone marrow and peripheral blood as well as other tissues. It activates STAT3 in peripheral blood cells, renal cortex, and glomeruli, alters IgG expression in B cells, increases glomerular *P*_alb_, and activates cultured podocytes. It causes albuminuria in mice and results in early focal glomerular scarring during chronic infusion. At least some of these effects are dependent on activation of the canonical JAK/STAT pathway. We interpret the increase in *P*_alb_ and activation of podocytes by CLCF-1 as well as increased renal cortical and glomerular pSTAT as evidence that CLCF-1 may play a role in FSGS. The finding that a specific inhibitor of JAK2 activation protects *P*_alb_ is consistent with a central role for the JAK/STAT pathway in early glomerular responses. We propose that alterations in immune cell function and elevation of specific autoantibodies may also contribute to the syndrome of FSGS and to its posttransplant recurrence. The data in this report suggest an injurious rather than a protective role for CLCF-1 and related cytokines and open new areas of investigation related to cytokine function and cellular injury and to the etiology and progression of renal disease and to the potential for novel targeted therapy for FSGS in selected patients.

## Abbreviations

ACR: Albumin: creatinine ratio
CLCF-1: Cardiotrophin-like cytokine factor-1
CNTF: Ciliary neurotrophic factor
CNTFR*α*: Ciliary neurotrophic factor receptor *α*
sCNTFR*α*: Soluble ciliary neurotrophic factor receptor *α*
CRLF1: Cytokine receptor-like factor-1
FSGS: Focal segmental glomerulosclerosis
gp130: Glycoprotein 130
IL-6: Interleukin-6
IL12: Interleukin-12
JAK: Janus kinase
LIFR*β*: Leukemia inhibitory factor receptor-*β*
*P*_alb_: Glomerular albumin permeability, measured during* in vitro* studies
SOCS: Suppressors of cytokine signaling
STAT: Signal transducer and activator of transcription
TNF*α*: Tumor necrosis factor-*α*.
